# Truncal asymmetry in school children: the effect of the parental age at birth

**DOI:** 10.1186/1748-7161-8-S1-O1

**Published:** 2013-06-03

**Authors:** TB Grivas, C Mihas, C Mazioti, S Sakellaropoulou, N Zisis, A Akriotis, RG Burwell

**Affiliations:** 1 Department of Trauma and Orthopaedics, "Tzanio" General Hospital of Piraeus, Piraeus, Greece; 2 General Hospital of Kimi, Kimi, Greece; 3 "Tzanio" General Hospital of Piraeus, Piraeus, Greece; 4 "Tzanio" General Hospital of Piraeus, Greece; 5 Centre for Spinal Studies and Surgery, Nottingham University Hospitals Trust, Queen's Medical Centre Campus, Nottingham, UK

## Background

Truncal back shape asymmetry (TBSA), as thoracic and/or lumbar humps, is the main indicator for referral to clinics during school-screening for IS, and also the most important sign for assessing IS. It is reported that maternal, but not paternal, age at birth is a risk factor for progressive IS.

## Aim

This report assesses the relation of parental age at birth to the development of TBSA in school children, which has not been evaluated yet.

## Methods

11,832 (5,855 males, 5,977 females) children and adolescents (5-17 years old, mean age: 11.34±2.79) were screened at their school for TBSA and/or scoliosis. The Prujis scoliometer was used to examine the students in standing, and sitting, forward bending positions. If at least one of the child's measured angles was equal to or exceeded 6 or 7 degrees of scoliometer reading, it was labelled as "Asymmetry 6" and "Asymmetry 7" respectively. The age, standing height and body weight of the children and the parental age were also documented. The parental age at birth and children's BMI were subsequently calculated. Asymmetries were tested for correlation with parental age at birth, which was transformed to a categorical variable in 5-year intervals. Pearson's Chi-squared test for univariate and logistic regression for quantitative univariate and multivariate analysis were used. The SPSS and STATA v. 11.0 statistical packages were used.

## Results

Maternal age (MA): Asymmetry6 and asymmetry7, only in boys, tended to decrease significantly as mother's age at birth increased. The significant and inverse effect of MA at birth on the appearance of asymmetry remained, only in boys, after adjusting for the child's age and BMI. Similar findings were documented for paternal age.

## Conclusions

The findings of this report indicate that parental age may possibly influence the presentation of TBSA in males, and possibly also in females, but, unexpectedly, by younger more than older mothers. The importance of the present findings is based on the premise that the intrauterine environment is crucial for programming the fetus for various health and disease outcomes throughout life. We suggest the mechanism involves an environmental factor, and by implication, epigenetics.
